# Letter to Editter

**DOI:** 10.1111/iwj.14037

**Published:** 2022-11-24

**Authors:** Meng‐Yuan Zhang, Yue‐Sheng Huang

**Affiliations:** ^1^ Department of Wound Repair Hospital of Southern University of Science and Technology Shenzhen PR China

Dear Editor in Chief,

We read the article *Methylene blue staining: A novel application to identify the damaged tissues on the surface of pressure ulcers* in detail and realised that methylene blue could be used to identify dead or damaged tissue on pressure ulcers, but we also found that there were few questions to be solved.

Firstly, the number of cases in this study was relatively small, which could reduce the credibility of the article. It is expected that the authors could increase the sample size in future studies.

Secondly, the article introduced the application of methylene blue to the wounds of pressure ulcers, but there was no classification to explain the clinical situations in which methylene blue was applied to the wounds. For example, whether it can be applied to all wounds, especially the identification of necrotic tissue in acute wounds. If possible, the precautions given in the application of methylene blue in acute wounds will be of key help to our clinical practice.

Thirdly, if we want to determine whether it is necrotic or damaged tissue, then methylene blue needs to be applied to all tissues, so it is very necessary to propose the recommended method of methylene blue in the article. In addition, we also hope to explain the commonly used methods in the recommended methods. The description should also include the concentration of methylene blue that can accurately infiltrate the tissue and the method to ensure the infiltration of methylene blue in place.

The last one is follow‐up treatment, that is to say, after using methylene blue for 24 hours, we hope that the author can explain different wound closure methods applicable to the wound.

If the author can answer the above questions, it will undoubtedly improve the overall quality of the article and attract more readers.

## Data Availability

Data sharing is not applicable to this article as no new data were created or analyzed in this study.

