# Erratum

**DOI:** 10.1111/iwj.14201

**Published:** 2023-08-09

In the article [1], there was a typesetting error in the title.

The incorrect title read:

Letter to Editter

The correct title should read:

Comment on “Methylene blue staining: A novel application to identify the damaged tissues on the surface of pressure ulcers”.

The online version has been corrected. We apologize for this error.
